# Proton NMR characterization of intact primary and metastatic melanoma cells in 2D & 3D cultures

**DOI:** 10.1186/s40659-017-0117-8

**Published:** 2017-03-16

**Authors:** Gokula Krishnan Ramachandran, Chen Hua Yeow

**Affiliations:** Department of Biomedical Engineering, National University of Singapore, E1-08-016, 9 Engineering Drive 1, Singapore, 117575 Singapore

**Keywords:** Nuclear magnetic resonance, Proton magnetic resonance spectrometry, Melanoma, Cancer

## Abstract

**Objective:**

To characterize the differences between the primary and metastatic melanoma cell lines grown in 2D cultures and 3D cultures.

**Methods:**

Primary melanoma cells (WM115) and metastatic melanoma cells (WM266) extracted from a single donor was cultured in 2D as well as 3D cultures. These cells were characterized using proton NMR spectrometry, and the qualitative chemical shifts markers were identified and discussed.

**Results:**

In monolayer culture (2D), we observed one qualitative chemical shift marker for primary melanoma cells. In spheroid cultures (3D), we observed nine significant chemical shifts, of which eight markers were specific for primary melanoma spheroids, whereas the other one marker was specific to metastatic melanoma spheroids. This study suggests that the glucose accumulation and phospholipid composition vary significantly between the primary and metastatic cells lines that are obtained from a single donor and also with the cell culturing methods. 14 qualitative chemical shift markers were obtained in the comparison between monolayer culture and spheroids cultures irrespective of the differences in the cell lines. Among which 4 were unique to monolayer cultures whereas 10 chemical shifts were unique to the spheroid cultures. This study also shows that the method of cell culture would drastically affect the phospholipid composition of the cells and also depicts that the cells in spheroid culture closely resembles the cells in vivo.

**Conclusion:**

This study shows the high specificity of proton NMR spectrometry in characterizing cancer cell lines and also shows the variations in the glucose accumulation and phospholipid composition between the primary and metastatic melanoma cell lines from the same donor. Differences in the cell culture method does plays an important role in phospholipid composition of the cells.

**Electronic supplementary material:**

The online version of this article (doi:10.1186/s40659-017-0117-8) contains supplementary material, which is available to authorized users.

## Background

14.1 million new cancer cases are reported every year with 8.2 million annual deaths [1]. As cancer is very heterogeneous, it is indispensable to have better characterization procedure for its diagnosis and treatment. One non-destructive way of characterizing cancer cells is by employing nuclear magnetic resonance. In this study, we intend to access the specificity of the proton NMR method using the two closely associated melanoma cell lines. Cancer cells contain higher levels of phospholipids than normal cells, which is responsible for enhanced cell proliferation and signal transduction. The alteration of phospholipid composition aids the cancer cells in invasion, metastasis and expression of growth factor receptors [2, 3]. Past studies concluded that ^1^H MRS could be used to diagnose cancer and also to monitor treatment responses [4–7]. It had been reported in gene expression studies that 576 genes were differentially expressed between primary melanoma and metastatic melanoma among which most of the genes showed decreased expression in metastatic melanoma when compared to primary melanoma [8]. These genes were found to be involved in cell adhesion, tumor suppression, cell cycle regulation and apoptosis. It is noteworthy that two proteins namely MAGEC1 (Melanoma antigen family c1) and FCRL1 (Fc receptor-like 1), which were known for melanoma progression were up-regulated in metastatic melanoma [8]. Another study showed that in primary melanoma SPRR1A/B, KRT16/17, CD24, LOR, GATA3, MUC15, and TMPRSS4 were up-regulated whereas, MAGE, GPR19, BCL2A1, MMP14, SOX5, BUB1, RGS20 were up-regulated in metastatic melanoma [9].

Coherent anti-Stokes Raman Scattering imaging revealed that free fatty acids (FFA) induced lipid accumulation in the cancer cells. FFA accumulation could adversely affect the cell–cell contact and promotes tissue invasion and positively correlated with the extend of metastasis [10]. Inhibition of the lipogenesis either by pharmacological drugs or using antisense shRNA resulted in the inhibition of tumor growth and metastasis after anti-angiogenic treatment [11, 12].

The higher levels of the choline related compounds were not only due to the biosynthetic pathways but also due to oncogene-induced activation of phosphatidyl choline and phosphatidyl ethanolamine-specific phospholipases. This resulted in the accumulation of choline related compounds in tumorous or actively proliferating cells [13]. Differences in the concentration of choline-containing compounds could be considered as an indicator for accessing the clinical response of cancer to chemotherapy. The outcome of the chemotherapy could be considered as positive if the choline-containing compounds decrease after the chemotherapy [14]. It is reported that metastatic melanoma of lymph node can be detected using proton NMR spectroscopy with a sensitivity of 92.9% and specificity of 90.3% [15]. Chemical shifts of choline could alone separate the melanoma biopsies from non-melanoma biopsy with an accuracy of 69% [16]. Another study reported elevated levels of choline, taurine, lactate and amino acids like alanine, lysine, glutamine and glutamate in biopsies could be potential biomarkers for the identification of both primary and secondary melanoma cells [17]. From these studies, it is evident that proton NMR spectroscopy is capable enough in discriminating melanoma cells from non-melanoma cells.

The biomarkers identified by the MRI coupled with ^1^H-MRS and proton nuclear magnetic resonance spectrometry (^1^H-NMR) could be correlated, as both these methods evolve from the same principle where the former was the addition of imaging to the later [18]. In a recent study, proton nuclear magnetic resonance spectrometry identifies a unique chemical shift biomarker 1.28 ppm for the identification of the neural stem and progenitor cells (NPC). This method was used to identify the NPC in the live mouse using the same biomarker under proton magnetic resonance spectroscopy (^1^H-MRS) [19]. From the previous studies, it is clear that magnetic resonance can be employed for cancer diagnosis using the lipid signals, but the specificity of the magnetic resonance method in detecting cancerous tissues is still not well established.

We hypothesize that the proton NMR method has high specificity and capable of distinguishing biologically related cell lines. To test this hypothesis, we explore the specificity of proton NMR method by characterizing the primary and metastatic melanoma cells that were extracted from a single donor. The specimens we selected for this study were WM115 (primary melanoma) and WM266 (metastatic melanoma) cell lines. These cells are closely related as they are excised from the same donor and vary only in the stages of cancer. We also explored the differences in these cells grown as adherent cells and as multi-cellular tumor spheroids using proton NMR. If our hypothesis is correct, then it is evident that proton NMR shows high specificity and suitable for cellular level characterization.

## Methods

### Cell culture

WM-115 (human primary melanoma) and WM-266 (human metastatic melanoma) cells were purchased from American Type Culture Collection (ATCC), USA. These cells were cultured in eagle’s modified essential medium (EMEM) with 10% fetal bovine serum (FBS). For the comparison of different cell lines in vitro by nuclear magnetic resonance (NMR), it is necessary that the cells should have the same histologic origin and same growth medium [3]. All the experiments were carried out in triplicates for both the cell lines.

### Extraction of the intact cells

The cells were cultured until they reached 90–95% confluence. The cells were washed twice with DPBS and harvested by trypsinization. The viability of the cells was checked by hemocytometer using bromophenol blue dye exclusion as a criterion for distinguishing live and dead cells. The samples with over 95% of viable cells were assumed to be suitable for the NMR spectrometry. The cells were diluted so that each sample has 1 × 10^7^ cells.

### Formation of cellular spheroids

**Fig. 1:**
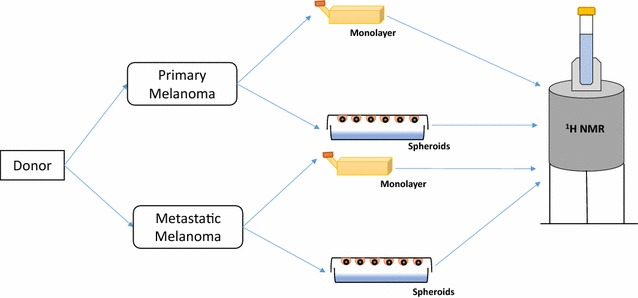
Experimental design

For the spheroids formation, the cells were grown under 2D culture condition and harvested using trypsinization. The harvested cells were checked for viability as described above. These cells were diluted into 2.5 × 10^6^ cells/mL and each sample is an aliquot of 4 mL containing 1 × 10^7^ cells. The diluted cell samples were drawn into 20 μL droplets on the lid of the petri dish. The petri dish was filled with 10 mL of DPBS which forms the hydration chamber. The petri dish lid was gently inverted and placed in the incubator for the spheroid formation. The petri dish was then incubated for 3 days. After 3 days, the spheroids could be observed by the naked eye as white spheres. The spheroids were harvested and 600 μL of D_2_O was poured gently on the harvested spheroids and carefully poured into the NMR tubes (Fig. 1).

### Nuclear magnetic resonance (NMR) spectrometry

We performed one-dimensional ^1^H NMR spectrometry (DRX500, Bruker USA) on the collected samples and matched controls using deuterated water (D_2_O) as the proton NMR solvent (Additional file 1: Figure S1). The acquired proton NMR spectral data was Fourier-transformed followed by phase and baseline correction using the Bruker XWinNMR software version 3.5. All the spectrums were aligned using the Mnova 11 (Mestrelab Research, Spain) software. The visible peaks were manually picked and peak lists were extracted for the metabolite identification. The peak list was given as input to the Metaboanalyst 3.0 online statistical software (http://www.metaboanalyst.ca/, Canada) for statistical analysis [20].

## Results

### NMR spectrum of the melanoma cells from monolayer culture and spheroid culture

The peaks were binned together by the moving window of 0.03 ppm and a step of 0.015 ppm, which results in a group of 131 chemical shifts. The binned data was then normalized using generalized log transformation and auto scaling.

### Chemical shift markers identified using proton NMR analysis

One-way ANOVA was performed for the identification of the statistically significant chemical shifts (*p* < 0.05). Among the significant chemical shifts (*p* < 0.05) markers, we focus only on the qualitative markers as they are more relevant for the clinical translation of this study. In the monolayer culture, the ^1^H NMR spectrums of the primary and metastatic melanoma cells were almost similar and varied only in their intensity (quantitative markers) expect the chemical shift at 5.86 ppm.

**Fig. 2:**
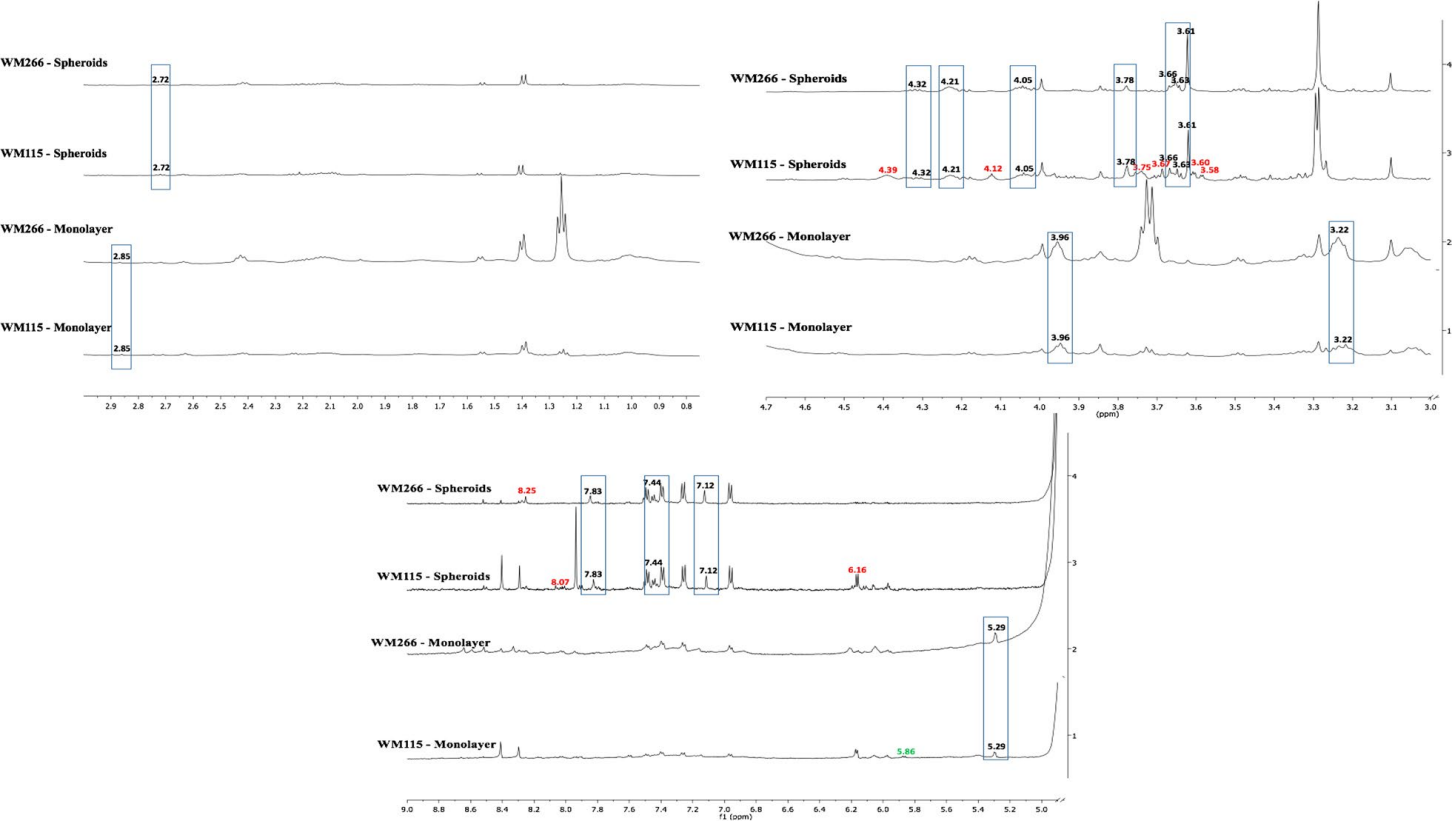
1D ^1^H NMR spectra showing the qualitative chemical shift markers; *red font* qualitative chemical shift markers between WM115 spheroids and WM266 spheroids; *green font* qualitative chemical shift markers between WM115 monolayer and WM266 monolayer; *black font–boxed* qualitative chemical shift markers between monolayers and spheroids irrespective of the type of cell lines

In the spheroid culture, we had observed several qualitative chemical shift markers for both primary and metastatic melanoma spheroids. Eight (3.58, 3.60, 3.67, 3.75, 4.12, 4.39, 6.16, 8.07 ppm) chemical shift markers were observed in the primary melanoma spheroids and one marker namely 8.25 ppm for metastatic melanoma spheroids (Fig. 2; Table 1).

**Table 1:** List of qualitative chemical shift markers and its chemical shift assignment

S. no	Cell line/culture type		^1^H NMR markers (ppm)	Assignment	Reference
1	2D	Primary melanoma	5.86 (d)	Guanosine	[21]
2		Metastatic melanoma	–	–	–
3	3D	Primary melanoma	3.58 (d)	Threonine	[22, 23]
			3.60 (d)	Threonine	[24]
			3.67 (s)	Glucose	[23]
			3.75	Glutamine	
			4.12 (t)	Glycerophospoethanolamine	[25, 26]
			4.39 (s)	*N*-Acetylgalactosaminitol	[27]
			6.16 (d)	Glucose	[28]
			8.07 (s)	NH_2_	[29]
4		Metastatic melanoma	8.25 (s)	ATP	[30]
5	Primary melanoma	2D	1.16 (s)	Lipids	[31]
			2.71 (s)	Dimethylamine	[32]
			2.74 (s)	Dimethylamine	[33]
			3.04	Creatine/phosphocreatine	[25] [34, 35]
			3.25 (s)	Choline head groups of phospholipids	[36]
6		3D	2.19 (s)	Adipate	[37]
			2.72 (t)	Unassigned	
			2.93 (s)	Dimethyl glycine	[26]
			3.58 (d)	Threonine	[23]
			3.61 (d)	Glycerol	[38]
			4.02 (s)	2 Hydroxyglutarate	[39, 40]
			4.12 (t)	Glycerophosphoethanolamine	[38]
			4.39 (s)	*N*-Acetylgalactosaminitol	[27]
			6.07 (d)	Unassigned	
			6.17 (s)	Unassigned	
7	Metastatic melanoma	2D	3.08	Methyl lysine	[40] [41]
			3.72–3.75 (q)	Unassigned	
			3.90 (s)	Glucose	[42]
8		3D	3.19 (s)	*O*-Acetylcarnitine	[37]
			3.48 (s)	Unassigned	
9	2D		2.85 (s)	Asparagine	[43]
			3.22	Choline	[25]
			3.96 (t)	β-Cyclodextrin	[44]
			5.29 (s)	Hc–ch (l, ch)	[31]
10	3D		2.72 (t)	Unassigned	
			3.63 (s)	Glycerol	[38]
			3.66	Glycerol/inositol	[38] [45]
			3.78	Phosphoinositol	[45]
			4.05 (t)	Ethanolamine	[45]
			4.21	Phosphocholine	[38]
			4.32	Glycerophosphocholine	[31]
			7.12 (s)	Ar–H	[31]
			7.44 (d)	Unassigned	
			7.83 (s)	Amide (–NH)	[37]

**Fig. 3:**
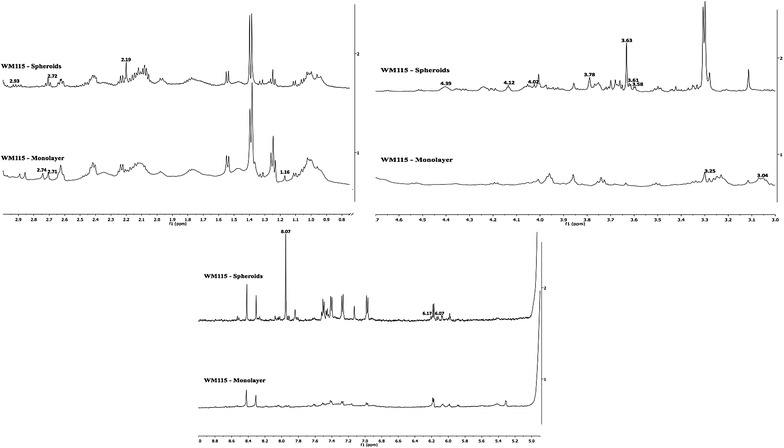
1D ^1^H NMR spectra showing the qualitative chemical shift markers between WM115 monolayer and WM115 spheroids

Melanoma cells were observed to behave differently with different cell culture methods. In this analysis, we identified the chemical shifts that were unique to the cell line by excluding the chemical shifts that were specific to the cell culture method, irrespective of the cell lines. Comparison of primary melanoma cells cultured as monolayer and as spheroid yields five chemical shift markers (1.16, 2.71, 2.74, 3.04, 3.25 ppm) for primary melanoma cells cultured as monolayer and 10 chemical shift markers (2.19, 2.72, 2.93, 3.58, 3.61, 4.02, 4.12, 4.39, 6.07, 6.17 ppm) for primary melanoma cells cultured as spheroids (Fig. 3).

**Fig. 4:**
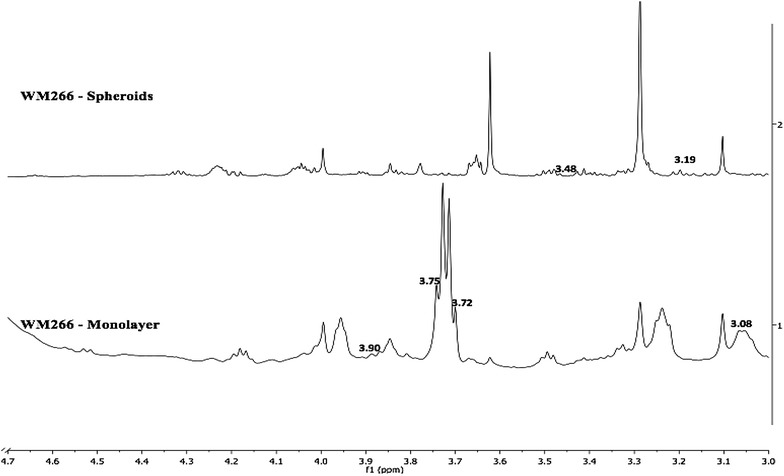
1D ^1^H NMR spectra showing the qualitative chemical shift markers between WM266 monolayer and WM266 spheroids

Similarly, in the case of metastatic melanoma cells, three chemical shift markers (3.08, 3.72–3.75, 3.90 ppm) were observed for metastatic melanoma cells cultured as monolayer and two (3.19 and 3.48 ppm) chemical shift markers were observed in metastatic melanoma cells cultured as spheroids (Fig. 4; Table 1).

In this study, we also intend to identify chemical shift markers with respect to the culturing method irrespective of the type of cell line. We were able to identify four chemical shift markers (2.85, 3.22, 3.96, 5.29 ppm) found only in the cells grown as monolayer and 10 chemical shift markers (2.72, 3.63, 3.66, 3.78, 4.05, 4.21, 4.32, 7.12, 7.44, 7.83 ppm) found specific to the cells grown as spheroids (Table 1).

**Fig. 5:**
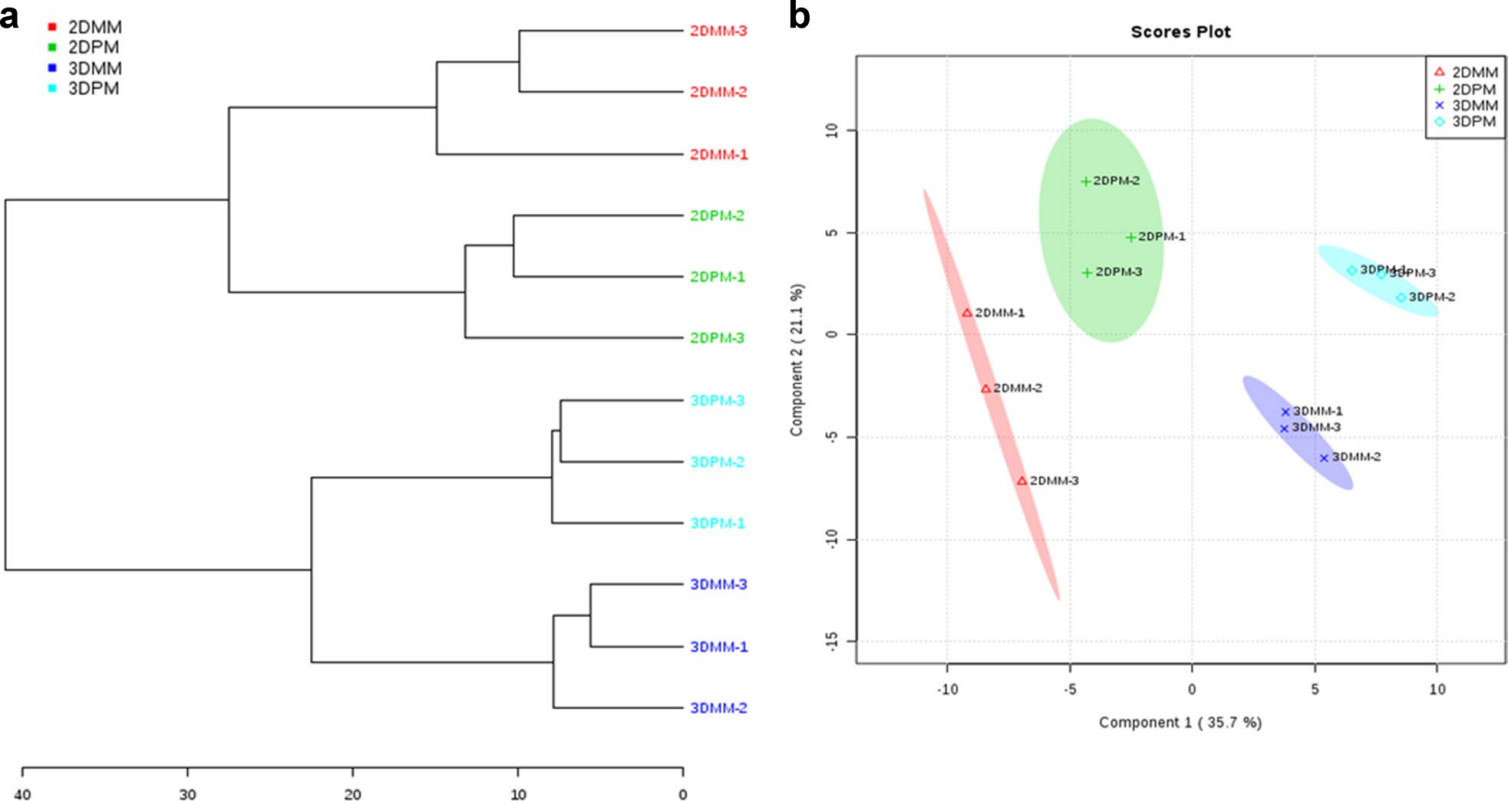
**a** Dendrogram showing distinct clusters of primary and metastatic melanoma cells cultured as monolayer and spheroids. This shows the impact of the cell culturing method. **b** PLS-DA plot showing the variance between the primary (PM) and metastatic melanoma (MM) cells cultured as monolayer and spheroids

Partial least square-discriminant analysis (PLS-DA) and clustering analysis performed between the primary and metastatic cells showed that culturing method had a more dominant influence over their ^1^H NMR spectrum than the cell line differences (Fig. 5).

## Discussions

### Guanosine as the biomarker for primary melanoma in monolayer culture

This chemical shift 5.86, which was unique to the primary melanoma cells cultured as monolayer is assigned to guanosine, which is the integral part of the cGMP, an important second messenger in various intra-cellular signal transduction pathways. This suggests that under monolayer culture, guanosine is the biomarker of primary melanoma cells (WM115).

### Primary melanoma spheroids and metastatic spheroids vary in glucose accumulation and phospholipid composition

The identified chemical shift markers of primary melanoma spheroids were assigned to threonine (3.58, 3.60 ppm), glucose (3.67, 6.16 ppm), glycerol-phosphoethanolamine (4.12 ppm) *N*-acetyl-galactosaminitol (4.39 ppm) and NH_2_ proton (8.07 ppm) (Table 1). The chemical shift marker of the metastatic melanoma spheroids was assigned to ATP (8.25 ppm). The primary melanoma spheroids suggest the presence of amino acid threonine which could indicate the presence of a membrane protein that was rich in threonine. Threonine had been reported in the literature as a biomarker for ER (endocrine hormone receptor) positive breast cancer [23]. Interestingly primary melanoma spheroids had been found to have glucose, a key energy source that was not detected in metastatic melanoma spheroids. It had been shown that during the detachment of cells from the extracellular matrix, the cancer cells stopped the glucose consumption that resulted in energetic stress. In melanoma, it was shown that during metastasis the ROS levels increases dramatically, which could be attributed to the cessation in the glucose uptake. When the glucose consumption was stopped the ATP production would cease, but we observed ATP as the biomarker for metastatic melanoma, this is because once the ATP level starts to decline AMP-activated protein kinase (AMPK) will be activated and inhibits fatty acid synthesis and activate fatty acid oxidation pathway. This shift in the fatty acid metabolism resulted in the generation of NADPH and promotes the generation of α-ketoglutaric acid from isocitric acid, which also increased the NADPH. This NADPH which is generated by the AMPK activation could enter into electron transport chain resulting in the synthesis of ATP [46]. *N*-Acetylgalactosaminitol, observed in the primary melanoma spheroids, forms an integral part of cancer associated glycoproteins [50].

### Differences in the cell culturing method are primarily reflected in the phospholipid composition

The comparison of the chemical shifts of the cells cultured as monolayers and cells cultured as spheroids irrespective of the cell line differences we could observe chemical shift signatures for both monolayer cultures as well as the spheroid cultures. The chemical shift signatures of the monolayer cultures were assigned to Asparagine (2.85 ppm), choline compounds (3.22 ppm), β-cyclodextrin (3.96 ppm), and lipids (5.29 ppm). From our data, the presence of lipids and choline compounds, which formed the plasma membrane were evident in the monolayer cultures as more cellular surface area had been exposed compared to the cells in the spheroid culture. The triplet at 3.96 is assigned to β-cyclodextrin, but the biological origin of this compound is unclear. They have a very interesting property of forming inclusion complexes with various lipophilic compounds [44].

On the other-hand the chemical shift signatures of the spheroids were assigned to glycerol (3.63 and 3.66 ppm), inositol (3.66 ppm), phosphoinositol (3.78 ppm), ethanolamine (4.05 ppm), phosphocholine (4.21 ppm), glycerophosphocholine (4.32 ppm) and amide group (–NH) (7.83 ppm). The presence of glycerol, inositol, ethanolamine, phosphocholine and glycerophosphocholine shows the existence of the glycerophospholipids, the key component of cell membrane suggests that the multicellular spheroids closely resembles the cells in vivo.

### Differences in the culturing method is reflected in the phospholipid of primary melanoma cells

Primary melanoma cells showed differences in their behavior with respect to the culturing method. The markers of primary melanoma cultured as monolayer were assigned to lipids (1.16, 5.29 ppm), dimethylamine (2.71, 2.74 ppm), phosphocreatine (3.04 ppm) and choline head groups of phospholipids (3.25 ppm). Thus primary melanoma cells in monolayer produce certain common cell membrane components like choline and lipids. They also contain phosphocreatine a key reserve for the high energy phosphates, which could be utilized for the formation of ATP. Dimethylamine could be the result of l-citrulline formation from asymmetric dimethyl arginine (ADMA) [48].

On the other-hand when these same primary melanoma cells were cultured as spheroids they produce some unique chemical shift signatures that could be assigned to adipate (2.19 ppm), dimethyl glycine (2.93 ppm), threonine (3.58 ppm), glycerol (3.61 ppm), 2-hydroxy glutarate (4.02 ppm), glycerophosphoethanolamine (4.12 ppm), N-acetylgalactosaminitol (4.39 ppm). Adipate is an acidity regulator, which plays a vital role in maintaining the acidity balance in the cellular microenvironment. This acidity regulation is very important as the cells would produce lactate as a result of their metabolic activity. Glycerol and glycerophosphoethanolamine were the integral components of the phospholipids in the cell membrane. This suggests that the 3D architecture of the multicellular spheroids of the metastatic melanoma cells vary from their monolayer counterpart primarily in the plasma membrane composition. 2-Hydroxy glutarate (2-HG) is a well known as oncometabolite which will usually accumulate in malignant cells due to the gain of function mutation of the isocitric dehydrogenases (IDH). Accumulation of the 2-HG inhibits the 2-oxo glutarate dependent oxygenases which in turn inhibits histone lysine demethylases thus impairing the epigenetic modifications of histones [49, 50]. This proves the closeness of the 3D spheroids with the in vivo tissues. Previous studies on the rectal adenocarcinoma had shown that *N*-acetylgalactosaminitol forms an integral part in all the four oligosaccharides isolated from the mucin-like glycoprotein of rectal adenocarcinoma. Clearly, *N*-acetylgalactosaminitol involves in the formation of cancer associated glycoproteins [47].

### Glucose accumulation and *O*-acetyl carnitine mark the differences between the metastatic melanoma cells cultured as monolayer and as spheroids

Variations in the chemical shift signatures with respect to the culturing method was also observed in the metastatic melanoma cells. The chemicals shifts of the monolayer cultured cells suggested the presence of methyl lysine (3.08 ppm), glucose (3.90 ppm), and whereas the chemical shifts of metastatic melanoma spheroids revealed the presence of *O*-acetyl carnitine (3.19 ppm). This data showed that methyl lysine and glucose resonating at 3.08 and 3.90 ppm respectively were the qualitative markers for the metastatic melanoma cells cultured as monolayer but the same cell line when cultured as spheroids they don’t produce chemical shift peaks at 3.08 and 3.90 ppm instead they produce a chemical shift at 3.19 which is assigned to *O*-acetyl carnitine. The chemical shift at 3.08 is attributed to proton from the CH_2_ group of the methyl lysine. Although it is previously reported that, this chemical shift manifested the presence of the SH_2_ group in taurine [51, 52]. In these studies, the 3.08 ppm chemical shift has appeared as a triplet, but in our data, the 3.08 ppm appear as a singlet. In a previous report, where the chromatin core particles where analyzed using proton nuclear magnetic resonance spectrometry encountered the 3.08 ppm chemical shift as a singlet, and it was assigned to the CH_2_ group proton of the methyl lysine [41]. Methylated amino acids especially methyl-lysine and methyl-arginine in the chromatin plays a vital role in recruiting proteins that induce structural changes in chromatin thus influencing gene expression and repression [53].

### Clinical projections of this study

In this study, we used the 3D spheroids, in order to minimize the limitations in its clinical translation. Flat monolayer cultures were the simplistic cancer models, having a physiologically uniform environment and lacked in the cell to cell attachment, which is not the case in the actual tumor environment. In in vivo tumors there exists cell to cell attachment, oxygen gradient, nutrition gradient and waste gradient. This behavior can be reproduced by culturing cells as 3D spheroids [54]. 2D flat monolayer cultured cells showed apical–basal polarity and lacks histological differentiation of the in vivo tumors, whereas histological morphology similar to the in vivo tumor can be achieved in 3D spheroids. [55, 56]. Like the in vivo tumors, 3D spheroids exhibit phenotypic heterogeneity in the cell proliferation rate, gene expression and differentiation which led to the heterogeneity in the function and morphology [57]. Few cells exhibited the stem cell-like characteristics such as self-renewal and undifferentiated multipotent phenotype called cancer stem cells (CSCs) [58]. These CSCs were observed in both in vitro 3D tumor spheroids and in vivo tumors [59], and these stemness related genes were found to be upregulated in 3D spheroids compared to the 2D monolayers [60]. All the above studies showed the cells grown as 3D spheroids resemble closely to the cells in the in vivo tumors. Thus, we expect there won’t be any potential limitations in the clinical translation of the results of this study and of course an actual clinical translational study is necessary to corroborate this statement.

## Conclusion

In this study, we showed that the two closely associated cell lines, which were obtained from the same donor and differed only in the stages of melanoma could be identified as different cell lines by employing 1D ^1^H NMR spectrometry. Thus proving the specificity of this method in characterizing cancer cell lines. Our study suggests that the glucose accumulation and phospholipid composition vary significantly between the primary and metastatic cells lines that were obtained from a single donor and also with the cell culturing methods. These results encourage further researches on understanding the effects of phospholipid and glucose accumulation in cancer development, progression and invasion. This study also showed that the method of cell culture would drastically affect the phospholipid composition of the cells and also depicts that the cells in spheroid culture closely resembled the cells in vivo.

## Additional file

**Additional file 1: Figure S1.** (A) 1D H^1^ NMR spectrum of media control for primary melanoma cells. (B) 1D H^1^ NMR spectrum of media control for metastatic melanoma cells. (C) 1D H^1^ NMR spectrum of spent media control for primary melanoma spheroids (WM115). (D) 1D H^1^ NMR spectrum of spent media control for metastatic melanoma spheroids (WM266). (E) 1D H^1^ NMR spectrum of trypsin. (F) 1D H^1^ NMR spectrum of DPBS.

## Abbreviations

NMR: nuclear magnetic resonance
1D: one dimensional
^1^H: proton
ATCC: American Type Culture Collection
FBS: fetal bovine serum
EMEM: Eagle’s modified essential medium
MAGL: monoacylglycerol lipase
FFA: free fatty acids
MAGEC1: melanoma antigen family c1
FCRL1: Fc receptor-like 1
FASN: fatty acid synthase
SREBPs: sterol regulatory element binding proteins
shRNA: short hairpin ribonucleic acid
PCA: principal component analysis
SPRR1A/B: small proline-rich protein 1A/B
KRT: keratin
LOR: loricrin
CD: cluster of differentiation
MUC: mucins
GATA: globin transcription factor
MAGE: melanoma antigen gene
GPR: G protein coupled receptors
TMPRSS4: transmembrane protease, serine 4
MMP: matrix metalloproteinases
SOX: SRY-related HMG box
BUB: budding uninhibited by benzimidazoles
RGS: regulators of G-protein signaling
BCL2A1: B-cell lymphoma 2-related protein A1
