# Supplementary material for: Proton NMR characterization of intact primary and metastatic melanoma cells in 2D & 3D cultures
Source: Biol Res. 2017 Mar 16;50:12. doi: 10.1186/s40659-017-0117-8 (PMC5353880; doi:10.1186/s40659-017-0117-8)
Supplement: Supplementary file 1 — Additional file 1: Figure S1. (A) 1D H1 NMR spectrum of media control for primary melanoma cells. (B) 1D H1 NMR spectrum of media control for metastatic melanoma cells. (C) 1D H1 NMR spectrum of spent media control for primary melanoma spheroids (WM115). (D) 1D H1 NMR spectrum of spent media control for metastatic melanoma spheroids (WM266). (E) 1D H1 NMR spectrum of trypsin. (F) 1D H1 NMR spectrum of DPBS. [file 40659_2017_117_MOESM1_ESM.docx]

**PROTON NMR CHARACTERIZATION OF INTACT PRIMARY AND METASTATIC MELANOMA CELLS IN 2D & 3D CULTURES**

Gokula Krishnan Ramachandran^1^ and Chen Hua YEOW^1^

^1^Department of Biomedical Engineering, National University of Singapore, Singapore.

**Supplementary Information:**


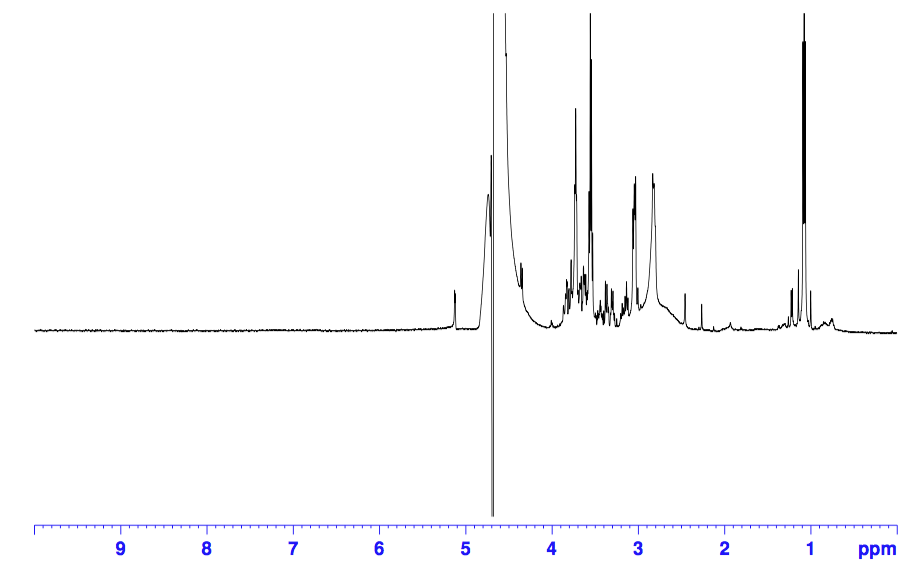

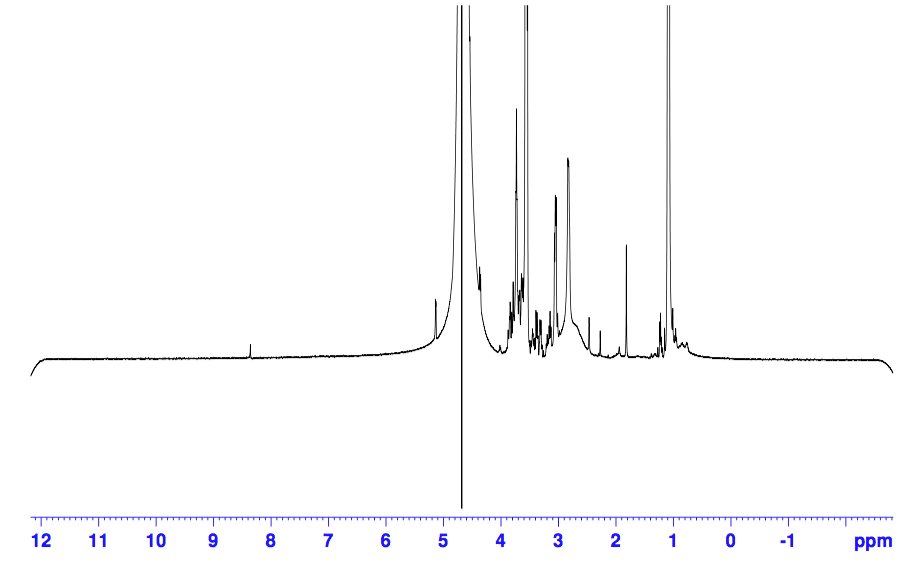


A

B


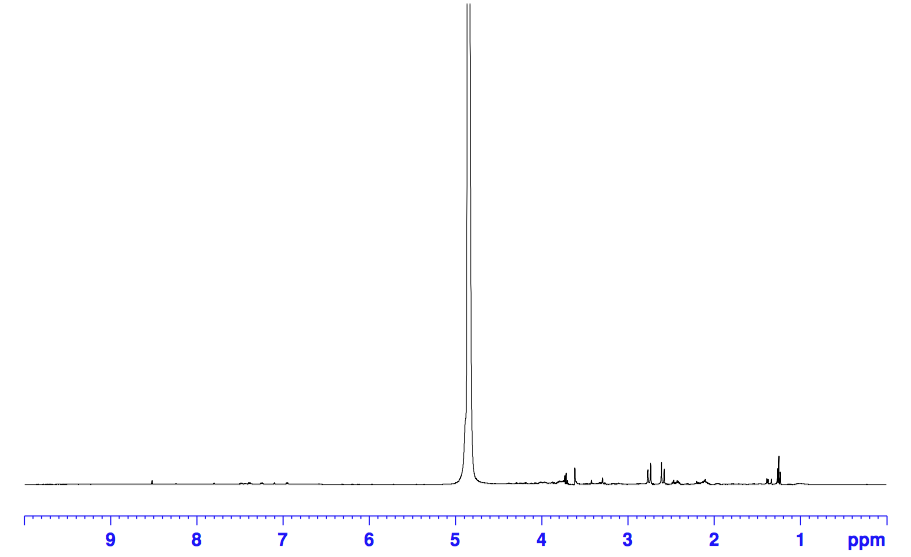

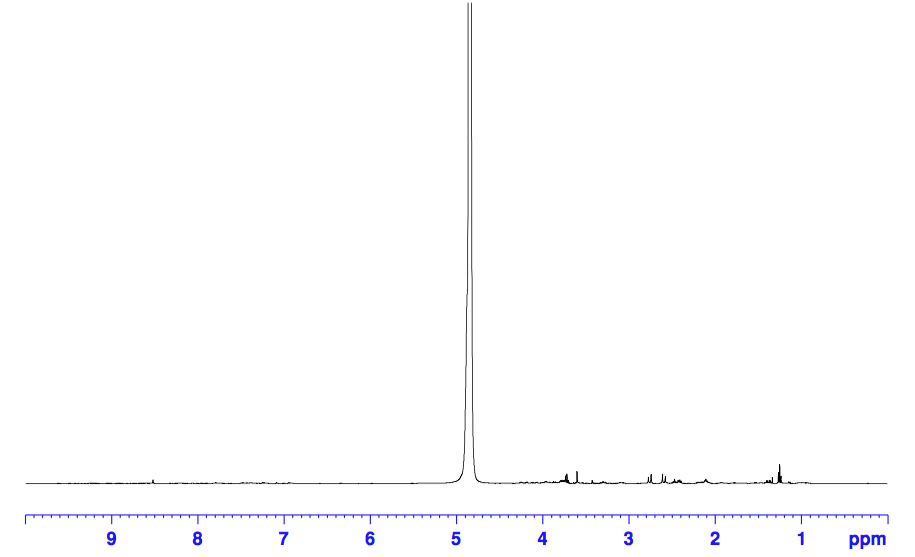


D

C


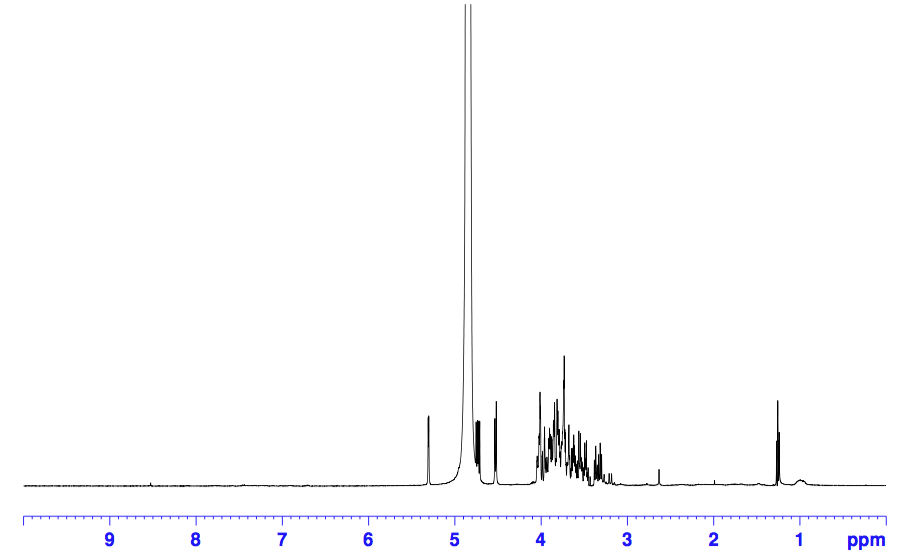

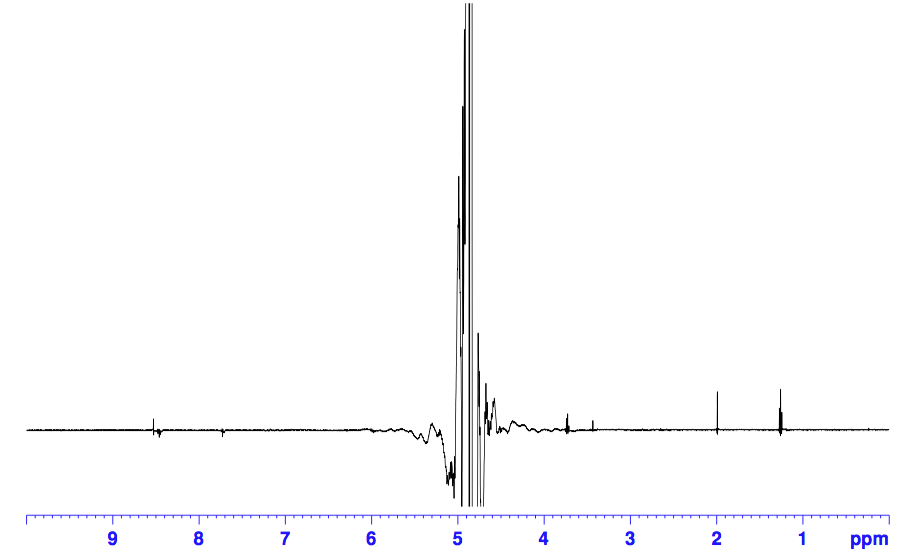


F

E

Figure S1: (A) 1D H^1^ NMR spectrum of media control for primary melanoma cells (B) 1D H^1^ NMR spectrum of media control for metastatic melanoma cells (C) 1D H^1^ NMR spectrum of spent media control for primary melanoma spheroids (WM115). (D) 1D H^1^ NMR spectrum of spent media control for metastatic melanoma spheroids (WM266). (E) 1D H^1^ NMR spectrum of trypsin. (F) 1D H^1^ NMR spectrum of DPBS.
